# Reporting and Methodological Observations on Prognostic and Diagnostic Machine Learning Studies

**DOI:** 10.2196/47995

**Published:** 2023-04-28

**Authors:** Khaled El Emam, William Klement, Bradley Malin

**Affiliations:** 1 School of Epidemiology and Public Health University of Ottawa Ottawa, ON Canada; 2 CHEO Research Institute Ottawa, ON Canada; 3 Department of Biomedical Informatics Vanderbilt University Nashville, TN United States

**Keywords:** reporting guidelines, machine learning, modeling studies, prognostic studies, methodological observations, diagnostic studies, ML models

## Abstract

Common reporting and methodological patterns were observed from the peer reviews of prognostic and diagnostic machine learning modeling studies submitted to JMIR AI. In this editorial, we summarized some key observations to inform future studies and their reporting.

## Introduction

The *JMIR AI* journal was launched at the beginning of 2022. During that first year, many of the papers submitted to the journal reported on prognostic studies that applied machine learning (ML) models. In this editorial update, we wish to highlight common patterns that were observed from the comments of the peer reviewers. Our objective in publishing this editorial is to inform authors about specific issues that should be documented and provide information about common methodological problems that can be avoided. Since these observations can help improve articles submitted to the journal, authors will benefit both in terms of acceptance rates and turnaround times for publication decisions. Furthermore, these observations may be of value to the broader ML community to inform the reporting of their studies. They are not intended to be comprehensive reporting guidelines but focus specifically on our observations with journal submissions.

We examined reviewers’ comments for papers submitted to *JMIR AI* over the entirety of 2022 (irrespective of their eventual publication decision). This included all papers remaining under review. We focused solely on papers that presented prognostic and diagnostic models using ML modeling techniques. The most common suggestions or critiques raised by reviewers were identified by counting observations in the reviewer comments. It was recognized that, at times, reviewers’ comments covered multiple overlapping issues or implied an issue without stating it completely. As a consequence, some judgment by us was required to decide which reviewer observations should be included in this update.

## Reporting and Methodological Observations

### The Degrees of Limitations

In some instances, there was a methodological weakness in the study. If this is raised by a reviewer, there is a tendency for authors to mention this issue in the “Limitations” section of the manuscript, rather than address it in the study itself. However, some weaknesses are not just standard limitations but affect the meaningfulness of the modeling that was performed and whether valid conclusions can be drawn from it. Not all weaknesses will be considered acceptable limitations, some of which we highlight throughout this article.

The limitations communicated in a manuscript present shortcomings due to practical or theoretical constraints presented to the model or algorithm, in which case it is anticipated that the constraints are out of the control of the authors and may inspire future research directions. As a hypothetical example, imagine that tissue samples are collected from donor lungs prior to lung transplantation, and a researcher subsequently develops a prognostic molecular test to predict if an adverse event will occur within the first 72 hours after lung transplantation surgery. This test is fundamentally limited to the molecular makeup of the donor because it neglects to consider the immunological response of the transplant recipient toward the prediction. In practice, surgical constraints prevent the collection of tissue samples immediately after transplantation.

In contrast, limitations by choice reflect decisions made in the scope, focus, methodology, and possibly aims of the study that can result in weaknesses that may be deemed unnecessary. The latter type of limitations needs to be addressed in the (re)submitted manuscript, which may require further analysis and rework. Of course, some judgment is necessary to distinguish between the two types of limitations, but the default of adding critical weaknesses to the “Limitations” section of a study report is not recommended.

### Documenting Reasons and Impacts of Data Sampling

Some studies start with a very large number of observations but end up using only a small proportion in the study. In many cases, the reduction in sample size is not an artifact of a random process. In such a case, it is possible that the authors have induced a selection bias in the data [1,2]. For example, if there are 1000 patient records with a particular diagnosis in a health care organization that meet the inclusion criteria, but only 500 are used in the study, how, if at all, does the subset differ from the initial larger group?

In some cases, missingness is a reason why many patients are excluded from an analysis. It is plausible that missing values of certain variables, which may include the outcome itself, may be correlated with specific groups of patients. Thus, the authors should try to explain how missingness affects patient characteristics. Could the patients with missing values be less severe cases and therefore the data set used to train a prognostic model consists of healthier patients? And, if this is the case, is the trained model capable of generalizing to the broader population when it is applied in practice?

### Avoiding Data Leakage

It is important to be cognizant of data leakage in model evaluation; otherwise, optimistic results may be obtained. An example of leakage is when there are multiple observations per patient distributed across the training and testing subsets of the data set. Effectively, information about the same patient may be included in both the training and testing data sets. Because the observations in the training and testing data sets are likely to be correlated, the error rate may be optimistic. Special care must be exercised to ensure that such leakage does not compromise the results of the analysis [2].

From a reporting perspective, authors should clarify if there are repeated or correlated observations, as well as the actions taken to avoid data leakage [3].

### Reporting Missingness and How It Is Handled

It is important to indicate how many observations were missing for each variable included in model building. If specific actions were performed to handle missingness, then these should be stated as well. For example, authors should report if a complete case analysis or a specific type of imputation was performed [3-5]. Moreover, if imputation methods are applied, then the affected variables and the imputation methods need to be reported and their parameterizations need to be described [4,6].

### Justifying the Choice of ML Model(s)

Justification of ML modeling techniques is a somewhat common reviewer comment regarding deficiencies in a manuscript. Some studies compare the performance of different types of ML models. In such situations, the selection of ML models should be justified [7-9].

Using logistic regression as a baseline is often a reasonable choice as it is a commonly used modeling method [10]. A recent systematic review showed that logistic regression performance is comparable to the use of ML models for clinical prediction workloads [11]. Therefore, it represents a realistic baseline workload. The choice of other methods should be justified. For example, it may be the case that an ML model is selected because it is commonly relied upon by the academic community or is a standard in practice. Moreover, it may be the case that a particular method is considered state of the art.

### Reporting Hyperparameter Tuning Methodology and Results

An ML algorithm is typically controlled by a collection of hyperparameters that influence how learning takes place. Authors should describe if any hyperparameter tuning was performed or if and what default parameters were used. If hyperparameter tuning was performed, then an explanation should indicate which method was applied (eg, grid search or Bayesian optimization), as well as what loss function was relied upon. If one or more models are being reported upon, then the final parameters should be included in the supplementary materials. An exception would be reasonable in the context of a simulation where thousands of models may be trained. In this case, a method indicating how the models are generated should be detailed to ensure reproducibility [3,7].

The method for evaluating the performance of the tuned model should also be described. For example, nested cross-validation would allow the performance to be computed on the tuned models. Then, the final set of hyperparameters is determined from a follow-up k-fold cross-validation, and these latter ones should be reported [8,9].

### Documenting the Decision Threshold

Studies that use classification or regression, where a decision threshold maps the classification scores to a class or category, are common. The decision threshold can have a big impact on the performance of the model [12,13], and the relative cost of incorrect decisions. The often-used default threshold of 0.5 is not always a good choice. Documentation of the threshold and justification for the value selected are necessary to enable the reader to properly interpret the model performance.

## Conclusions

While this summary pertains to prognostic and diagnostic models mostly for structured data, many of the points are relevant for other types of data modalities (eg, image processing). Moreover, it should be recognized that the observations covered in this editorial are not exhaustive as there are other subtle issues that are highlighted by reviewers for specific studies. Nonetheless, adhering to the reporting recommendations and methodological considerations indicated above will be beneficial for *JMIR AI* submissions.

## Abbreviations

ML: machine learning
